# Three-Dimensional Outcome Assessments of Surgical Correction in Cleft and Noncleft Patients with Class III Skeletal Relation: A Case-Control Study

**DOI:** 10.1155/2021/4572397

**Published:** 2021-08-13

**Authors:** Te-Ju Wu, Cheng-Chun Wu, Chi-Yu Tsai, Yi-Hao Lee, Yu-Jen Chang, Shiu-Shiung Lin, Jui-Pin Lai

**Affiliations:** ^1^Department of Orthodontics, Kaohsiung Chang Gung Memorial Hospital and Chang Gung University College of Medicine, Kaohsiung, Taiwan; ^2^Department of Plastic Surgery, Kaohsiung Chang Gung Memorial Hospital and Chang Gung University College of Medicine, Kaohsiung, Taiwan

## Abstract

**Background:**

The orthognathic strategies to treat patients with a concave profile but different tissue conditions remain controversial. The aim of this case-control study was to investigate the outcome predictability of orthognathic surgery in cleft lip and palate (CLP) patients and matched controls.

**Methods:**

Fifty consecutive CLP and 45 matched non-CLP patients who received whole-piece Le Fort I and bilateral sagittal split osteotomy to correct class III skeletal relations were enrolled. The outcome discrepancies (ODs) from simulations among all groups were evaluated with consideration of the possible influences from planned surgical movements (PSM). Receiver operating characteristic curves were used to determine threshold values of PSMs that yielded clinically relevant OD.

**Results:**

Unilateral CLP (UCLP) patients had comparable postsurgical OD to non-CLP patients in both jaws, whereas bilateral CLP (BCLP) patients had greater deviations from predicted results. Vertical movement of the A − point > 1.33 mm and yaw correction > 1.65° in the BCLP patients was associated with clinically relevant maxillary OD.

**Conclusions:**

The OGS outcomes of BCLP patients were less predictable than those of the UCLP and noncleft patients. Vertical movements of the A − point > 1.33 mm and yaw correction > 1.65° in BCLP patients increased OD to a clinically relevant extent.

## 1. Introduction

Orthognathic surgery (OGS) is the treatment of choice for patients with excessive skeletal discrepancies [1, 2]. Extraordinary midface retrusion is a well-recognized phenomenon in cleft lip and palate (CLP) patients. Given the desire to recover or enhance facial aesthetics, the predictability of surgical results is a strong concern for surgeons, orthodontists, and patients. However, OGS is usually more challenging in CLP patients than in non-CLP patients because of the remarkable postsurgical relapse [3, 4]. Although two-jaw surgery can provide functional harmony with correction of the maxillomandibular complex (MMC) [5], soft tissue tension and bony segment instability inherently influence the postsurgical stability of CLP patients [6].

Conventionally, it is assumed that the postsurgical changes would differ between CLP and non-CLP patients. Nevertheless, existing evidence did not fully support such an assumption [6, 7]. For instance, maxillary advancement, the major component in treatments of patients with class III jaw relation, is ranked as “stable” in bimaxillary procedures of non-CLP patients. However, the so-called “stable” procedure is associated with a diverse relapse rate ranging from 25% to 49% [8]. On the other hand, in CLP patients, a 37% rate of horizontal relapse was reported in an earlier review [9]. Such controversial results [8, 9] implied that the actual impacts from inherent tissue defects and strain of CLP patients were not clearly revealed.

There have been only a few case-control studies to investigate how tissue disharmony affects the orthognathic outcomes in CLP patients [6, 10]. Based on the postsurgical results of patients receiving only maxillary advancement, comparable relapse tendency was reported between unilateral CLP (UCLP) patients and noncleft patients [6]. On the other hand, with concomitant porous-block hydroxyapatite grafting, Mehra et al. also reported similar outcome predictability in two-jaw surgeries among CLP and non-CLP patients [10].

The controversial results against the common acknowledgements might result from the limitation of traditional cephalometric assessments. The surgical plans of CLP patients have been reported to be unable to completely fulfill the simulated goals intraoperatively by conventional two-dimensional evaluation [11]. Not until development of three-dimensional surgical simulation (3DSS) has the actual difference from the planned jawbone position been able to be determined [12, 13, 14].

Therefore, the aim of this study was to assess the OGS outcomes of CLP patients by using a case-control design. The central hypothesis was that there is no difference in the outcome predictability between CLP and non-CLP patients.

## 2. Materials and Methods

In this study, the medical records of 200 consecutive patients who underwent OGS from January 2013 to September 2017 at the Craniofacial Center of Kaohsiung Chang Gung Memorial Hospital (Kaohsiung, Taiwan) were retrospectively reviewed. A total of 45 healthy non-CLP adult patients with mandibular prognathism and 50 nonsyndromic CLP adult patients met the inclusion criteria for analysis. All patients underwent whole-piece Le Fort I osteotomy and bilateral segmental sagittal osteotomy (BSSO) to correct jawbone discrepancies with the use of 3DSS. Patients with syndromic craniofacial disorders and those who underwent multipieced maxillary osteotomy, posttraumatic reconstruction, facial reconstruction, or modified surgical planning intraoperatively were excluded. The study protocol was approved by the Institutional Review Board of Kaohsiung Chang Gung Memorial Hospital (approval no. 201701645B0).

### 2.1. Data Retrieval and Processing

All the images were retrieved from medical CT (Aquilion, Toshiba Corp., Tokyo, Japan) (120 kVp; 350 mA; rotation time, 0.5 s; slices thickness, 0.5 mm) three weeks before the OGS. The Rhinoceros 5.0 (Robert McNeel & Associates, Seattle, Wash.) and Geomagic Studio (12th edition; Geomagic, Inc., Cary, N.C.) were used for image processing and virtual planning. The tentative plans were validated by setting the final occlusion of MMC checked by senior orthodontists. The final orientation and feasibility of the whole planning were confirmed by the same surgeon (J.P. Lai).

### 2.2. Fabrication of the Surgical Guide (the Detailed Procedures Were Described in Reference [13])

**Figure 1:**
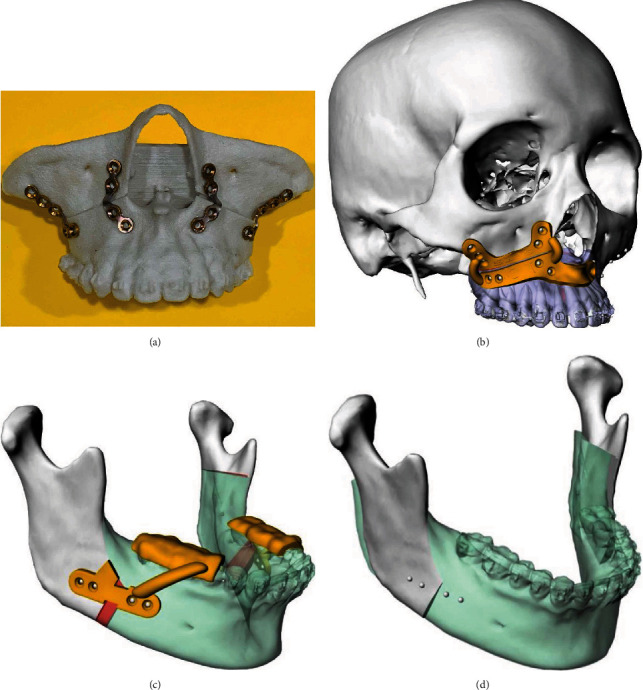
(a) Reverse engineering was applied to fabricate the surgical guide. A stereolithographic model demonstrating the planned maxillary reposition was produced. The fixation miniplates serving as the guiding plate (the 2nd guiding plate) were prebent according to the plate holes marked on the model. (b) Anterior nasal spine (ANS) and infrazygomatic crest (IZC) were used as reference structures for designing the guiding plate on the presurgical virtual maxilla. (c, d) The mandibular guiding plates were also fabricated according to the final position of MMC to provide predicted amounts of the movements of the mandibular segments intraoperatively.

The reverse engineering was applied to the fabrication of the surgical guide (Figure 1). A stereolithographic model demonstrating the planned maxillary reposition was produced (Figure 1(a)). The fixation miniplates serving as the guiding plate (2^nd^ guiding plate) were prebent according to the plate holes marked on the model (Figure 1(a)). Meanwhile, another guiding plate (1^st^ guiding plate) registering the orientation and thickness of the cutting lines was also 3D printed with clear biocompatible resin (MED610) (Figure 1(b)). On the other hand, the mandibular guide was also fabricated according to the final position of the MMC (Figures 1(c) and 1(d)).

### 2.3. The Surgical Procedures and Post-OGS Caring Protocol

All the patients received the “maxilla-first” concept for the surgical procedures. The first guiding plate was adapted to the maxillary surface to locate the screw holes and the cutting lines before the Le Fort I osteotomies (Figure 1(b)). After releasing the maxilla, the single stent technique was applied to guide the distal mandibular segments. At last, the prebent mandibular miniplates were used to verify the position of the proximal mandibular segments. All the surgical procedures have been performed by the same surgeon (JPL).

All the patients received the same postsurgical caring protocol including the intermaxillary fixation for 2–4 weeks and bilateral anterior vertical elastics for another 2-4 weeks. The postsurgical orthodontic treatments were initiated once primary wound healing was achieved.

### 2.4. Using Representative Triangles to Verify the Jawbone Changes

**Figure 2:**
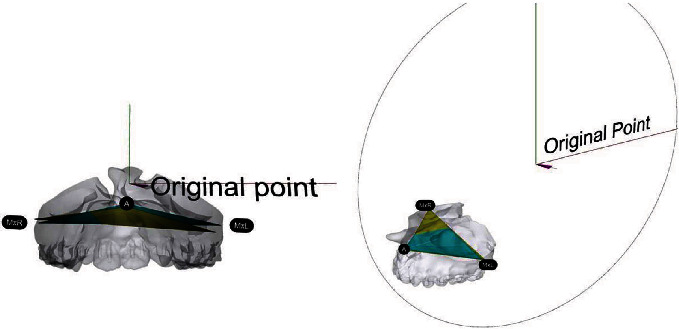
The virtual triangles representing orientation of the maxilla in different stages (yellow: simulation, blue: 6-month outcome) were used for the outcome assessments. All the three-dimensional images were registered on the same coordination system.

All the virtual planning was carried out on the world coordinate system. The virtual skulls were oriented by the reference plane passing through the bilateral orbitales and porions. The images of different stages were registered by the voxel-based method to determine the surgical movements. To verify the jawbone movements of each stage, a virtual triangle was plotted along with three bony anatomic end points including A-point and the most lateral points bilaterally, the MxR and the MxL (Figure 2). Once the A-point was registered by orthodontists, the tangent lines passing through the A-point were generated automatically to identify the MxR and the MxL at the same transverse plane. Such a representative triangle was then transferred to different stages by the voxel-based registration of the posterior nasal spine (PNS) to superimpose the virtual maxilla without deformation. More details have been described in our earlier study [12]. The jawbone orientations between different stages were then assessed by measuring the linear movements of each landmark and angular differences among the representative triangles.

### 2.5. The Cephalometric Assessments of Mandibular Position

The mandibular position was surveyed by lateral cephalometric films by the AudaxCeph Empower software (Version 5.2, Ljubljana, Slovenia). The distance from the pogonion to the nasion perpendicular line (A-Nv) was measured to assess the mandibular OD in the vertical direction. Meanwhile, the menton projection to the Nv was used to verify the mandibular position in the sagittal axis.

### 2.6. Reliability Test and Statistical Analysis

After collecting the primary data, 25 patients were randomly chosen for assessment of the interobserver and intraobserver reliabilities of the proposed method at a minimum interval of 2 weeks. The intraclass correlation coefficients were used to test the interobserver and intraobserver reliabilities of this method. The one-sample *t*-test was used to examine the positional differences of the virtual maxilla between the actual outcome and the simulation model (Table 1).

### 2.7. Identification of the Cutoff Values Leading to Outcome Discrepancies (OD) of Clinical Significance

Because the planned surgical movements (PSMs) of non-CLP and CLP patients might not be equivalent (Table 2), ANCOVA was chosen to adjust the mean value in each group before describing intergroup differences. Post hoc analysis (Scheffe method) was adopted to further identify intergroup differences (Table 3). For those measurements showing intergroup differences, receiver operating characteristic curves (ROC curves) were plotted to identify the cutoff values leading to OD of clinical significance (Table 4). At last, the one-way ANOVA was used to evaluate the mandibular OD among all groups.

## 3. Results

The one-sample *t*-test showed significant OD from simulation in all of the examined measurements in each group (Table 1). According to the results, the CLP usually required larger PSM than the non-CLP patients. However, there was no difference in PSM between the unilateral CLP (UCLP) and BCLP patients (Table 2).

The ANCOVA was then performed to adjust possible effects of PSM on OD. The results showed no significant difference in OD between the non-CLP and UCLP groups. On the other hand, three translational measurements (A-Z, MxR-Y, and MxL-Z) and one angular measurement (yaw) revealed significant intergroup differences. In these four measurements, the BCLP group showed increased maxillary OD than the UCLP and non-CLP groups (Table 3). The similar pattern was also revealed in the mandibular assessment (Table 5).

The ROC curves were then plotted to determine the cutoff values leading to 2 mm/2° OD. Because of the homogeneous characteristics, the non-CLP and UCLP groups were regarded as having the same characteristics. According to the results, the >1.33 mm anterior vertical movements (A-Z) and/or >1.65° yaw correction in BCLP are more vulnerable to OD of clinical significance (Table 4).

The intraexaminer and interexaminer reliabilities were in agreement (0.972 and 0.988, respectively).

## 4. Discussion

OGS is the treatment of choice for patients with excessive skeletal discrepancies [1, 2]. However, the surgical treatments of patients with craniofacial deformities are especially challenging [4]. Therefore, in the present study, we compared the outcome predictability between CLP and non-CLP patients in a case-control manner.

According to the results, the postsurgical outcomes were not identical to the presurgical simulation. All groups presented significant OD in all translational and angular measurements (Table 1). Such differences could be attributed to repositioning errors during operation and postsurgical relapse. In the present study, all patients received the same 3DSS protocol, surgical procedures, and guiding modalities, such that repositioning errors should have equally affected all groups. Therefore, postsurgical relapse may have been the major contributor to OD differences between the groups.

In addition to surgical procedures, which were controlled by enrolled criteria, PSMs are the other well-known factors of postsurgical relapse [12, 15]. In the present study, larger PSMs were prescribed to correct bony discrepancies in the CLP patients (Table 2). Under this circumstance, the actual effects of CLP-related deformities were masked. Therefore, the surgical predictabilities between the CLP and non-CLP patients were compared after statistically adjusting for PSM factors (Table 3). The results indicated that UCLP had a level of postsurgical OD comparable to that of non-CLP patients in all translational and angular measurements. However, the BCLP group is inherently more vulnerable to reduced surgical predictability in both jaws. Thus, the central hypothesis of this study was partially rejected. The results showed that UCLP patients had potentially equivalent OGS predictabilities to non-CLP patients, whereas BCLP tended to have larger discrepancies from the presurgical simulation estimates. This finding agreed with earlier cephalometric reports [6, 7]. Compared with UCLP patients, the unique characteristics, such as isolated premaxilla and bilateral alveolar clefts of BCLP patients, were believed to contribute to the instability [6, 7].

For decades, CLP patients were regarded as a special group because of their congenital deformities. However, according to recent reviews, non-CLP patients [8] did not obtain overwhelming advantages in postsurgical stability versus CLP patients [9]. The present study results support such an idea.

Generally, 2 mm differences have been commonly regarded as clinically relevant changes [11]. According to the results, vertical repositioning of the anterior maxilla > 1.33 mm in BCLP would result in a clinically relevant vertical OD. Additionally, for a 2° difference, the yaw corrections more than 1.65° would possibly face dominant OD after surgery (Table 4).

These results could be useful in guidelines for clinical practice. For CLP-related OGS, maxillary advancement with vertical downward movement has previously been reported to be unstable after surgery [16, 17]. The ROC results in the present study support such a concept, especially in BCLP patients. To improve vertical stability, the intraoperative grafting [18] and sufficient incisor display set up would be helpful in the BCLP patients. For yaw correction, small changes can noticeably affect postsurgical predictability in BCLP patients. To limit yaw correction when adjusting the MMC, orthodontists should try to achieve optimal coordination of dental and skeletal discrepancies before surgery.

There are some limitations in this study. First, although non-CLP patients experienced major relapse within the first 6 months after surgery [19, 20], the 6-month observation might not be long enough to reveal the progressed changes in CLP patients. Second, our study included only the CLP patients receiving whole-piece Le Fort I osteotomy, so we cannot provide insights regarding patients who received multipieced Le Fort I procedures. Finally, due to the lack of a reliable mandibular registration method, which is the common limitation of similar studies, only the cephalometric assessments could be provided for the mandibular assessments.

## 5. Conclusions

The OGS outcomes of BCLP patients are less predictable than those of UCLP and noncleft patients. Vertical movements of the A − point > 1.33 mm and yaw correction > 1.65° in BCLP patients increased OD to a clinically relevant extent.

## Figures and Tables

**Table 1:** The angular and translational outcome differences from simulation models of all samples (one-sample *t*-test).

Groups	Non-CLP (45)		BCLP (17)		UCLP (33)	
Outcome discrepancy	Mean	*P* value	Mean	*P* value	Mean	*P* value
Translational (mm)						
A-X	0.66 ± 0.53	0.000^∗^	0.69 ± 0.53	0.000^∗^	0.61 ± 0.48	0.000^∗^
A-Y	0.69 ± 0.73	0.000^∗^	1.15 ± 0.83	0.000^∗^	0.96 ± 0.89	0.000^∗^
A-Z	0.73 ± 0.66	0.000^∗^	1.38 ± 1.39	0.000^∗^	0.83 ± 0.68	0.000^∗^
MxR-X	0.61 ± 0.44	0.000^∗^	0.80 ± 0.66	0.000^∗^	0.71 ± 0.52	0.000^∗^
MxR-Y	1.08 ± 0.95	0.000^∗^	1.72 ± 1.14	0.000^∗^	1.06 ± 0.93	0.000^∗^
MxR-Z	0.99 ± 0.71	0.000^∗^	0.93 ± 0.70	0.000^∗^	1.08 ± 0.91	0.000^∗^
MxL-X	0.63 ± 0.48	0.000^∗^	0.84 ± 0.76	0.000^∗^	0.69 ± 0.49	0.000^∗^
MxL-Y	0.75 ± 0.75	0.000^∗^	1.31 ± 1.11	0.000^∗^	1.20 ± 1.14	0.000^∗^
MxL-Z	1.12 ± 0.74	0.000^∗^	1.62 ± 1.02	0.000^∗^	1.23 ± 0.80	0.000^∗^
Angular (°)						
Roll	0.94 ± 0.81	0.000^∗^	1.34 ± 0.88	0.000^∗^	1.16 ± 0.77	0.000^∗^
Pitch	1.97 ± 1.62	0.000^∗^	2.64 ± 2.86	0.000^∗^	2.34 ± 1.71	0.000^∗^
Yaw	1.07 ± 0.86	0.000^∗^	1.72 ± 1.13	0.000^∗^	1.13 ± 0.87	0.000^∗^

^∗^The mean difference is significant at the 0.05 level.

**Table 2:** Comparison of the PSMs of all samples (ANOVA).

	Groups			Scheffe			
Outcome discrepancy	Non-CLP (45)	BCLP (17)	UCLP (33)	*P* value	N^@^-B^#^	N-U^%^	B-U
Translational (mm)							
A-X	1.05 ± 0.91	1.56 ± 1.38	1.74 ± 1.37	0.032^∗^	-0.51	-0.69^∗^	-0.19
A-Y	3.22 ± 1.39	4.91 ± 1.91	4.26 ± 2.02	0.001^∗^	-1.69^∗^	-1.05^∗^	0.64
A-Z	1.43 ± 1.01	1.54 ± 1.95	1.13 ± 1.02	0.436	-0.11	0.30	0.41
MxR-X	0.73 ± 0.84	1.29 ± 1.55	1.28 ± 0.92	0.037^∗^	-0.55	0.55	0.006
MxR-Y	3.55 ± 1.69	4.84 ± 2.44	5.26 ± 3.25	0.009^∗^	-1.29	-1.71^∗^	-0.42
MxR-Z	2.82 ± 1.75	1.99 ± 2.66	2.14 ± 1.42	0.156	0.83	0.68	-0.15
MxL-X	0.72 ± 0.77	1.21 ± 1.61	1.20 ± 0.81	0.061	-0.49	-0.48	0.00
MxL-Y	2.82 ± 1.76	4.84 ± 2.09	3.94 ± 2.29	0.001^∗^	-2.02^∗^	-1.12	0.90
MxL-Z	2.88 ± 1.72	1.27 ± 0.69	2.05 ± 2.17	0.005^∗^	1.61^∗^	0.83	-0.77
Angular (°)							
Roll	1.30 ± 1.08	2.67 ± 4.93	2.10 ± 1.75	0.106	-1.37	-0.80	0.57
Pitch	4.25 ± 2.65	4.33 ± 3.69	2.29 ± 2.40	0.006^∗^	-0.09	1.96^∗^	2.04
Yaw	1.63 ± 1.74	2.09 ± 1.53	3.47 ± 2.98	0.002^∗^	-0.46	-1.84^∗^	-1.38

^∗^The mean difference is significant at the 0.05 level; ^@^Non-CLP patients; ^#^patients with bilateral cleft lip and palate; ^%^patients with unilateral cleft lip and palate.

**Table 3:** ANCOVA on outcome discrepancies and characteristics of the samples.

	Regression coefficients (*n* = 95)			
Outcome discrepancy	PSM	N^@^-B^#^	N-U^%^	B-U
Translational (mm)				
A-X	0.018	0.021	-0.066	0.087
A-Y	0.089	0.312	0.184	0.128
A-Z	0.017	0.642^∗^	0.097	0.545^∗^
MxR-X	0.079	0.146	0.060	0.086
MxR-Y	0.023	0.609^∗^	-0.062	0.671^∗^
MxR-Z	0.007	-0.053	-0.088	-0.142
MxL-X	0.170^∗^	0.128	-0.027	0.155
MxL-Y	0.144^∗^	0.271	0.290	-0.019
MxL-Z	0.082	0.627^∗^	0.181	0.445
Angular (°)				
Roll	0.107^∗^	0.259	0.138	0.121
Pitch	0.176^∗^	0.646	0.119	-0.062
Yaw	0.136^∗^	0.592^∗^	-0.193	0.785^∗^

^∗^The mean difference is significant at the 0.05 level; ^@^non-CLP patients; ^#^patients with bilateral cleft lip and palate; ^%^patients with unilateral cleft lip and palate.

**Table 4:** ROC curves were plotted to identify the cutoff value leading to ODs of clinical significance.

	AUROC	Best cutoff point	Sensitivity	Specificity	Accuracy
Abi_AZ					
Control/unilateral	0.684	1.37	100	47.9	52.6
Bilateral	0.810	1.33	100	71.4	76.4
Abi_LZ					
Control/unilateral	0.680	2.78	81.8	59.7	62.8
Bilateral	0.558	0.60	100	33.3	54.9
RY					
Control/unilateral	0.542	1.05	30.8%	95.4%	84.6%
Bilateral	0.667	7.31	60.0%	100%	88.2%
Abi_yaw					
Control/unilateral	0.678	2.0	76.9	60.0	62.8
Bilateral	0.857	1.65	100	70.0	82.4

**Table 5:** The cephalometric assessments of mandibular position.

	Groups			LSD		
Outcome discrepancy	Non-CLP (45)	BCLP (17)	UCLP (33)	N^@^-B^#^	N-U^%^	B-U
Pog-Nv (sagittal OD)	0.54 ± 2.65	4.30 ± 4.47	0.98 ± 3.83	-3.76^∗^	-0.44	3.32^∗^
Me on Nv (vertical OD)	−0.36 ± 1.74	−3.60 ± 2.53	−0.96 ± 2.20	-3.23^∗^	0.59	2.64^∗^

^∗^The mean difference is significant at the 0.05 level; ^@^non-CLP patients; ^#^patients with bilateral cleft lip and palate; ^%^patients with unilateral cleft lip and palate.

## Data Availability

The data used to support the findings of this study are included within the article.
